# Overexpression of Mechano-Growth Factor Modulates Inflammatory Cytokine Expression and Macrophage Resolution in Skeletal Muscle Injury

**DOI:** 10.3389/fphys.2018.00999

**Published:** 2018-07-26

**Authors:** Keng-Ting Sun, Kwok-Kuen Cheung, Shannon W. N. Au, Simon S. Yeung, Ella W. Yeung

**Affiliations:** ^1^Muscle Physiology Laboratory, Department of Rehabilitation Sciences, The Hong Kong Polytechnic University, Kowloon, Hong Kong; ^2^Centre for Protein Science and Crystallography, School of Life Sciences, The Chinese University of Hong Kong, Shatin, Hong Kong

**Keywords:** skeletal muscle injury, inflammation, muscle regeneration, insulin-like growth factor 1, mechano-growth factor, myeloid cells, macrophages, apoptosis

## Abstract

In muscle regeneration, infiltrating myeloid cells, such as macrophages mediate muscle inflammation by releasing key soluble factors. One such factor, insulin-like growth factor 1 (IGF-1), suppresses inflammatory cytokine expression and mediates macrophage polarization to anti-inflammatory phenotype during muscle injury. Previously the IGF-1Ea isoform was shown to be anti-inflammatory. Another isoform of IGF-1, mechano-growth factor (MGF), is structurally and functionally distinct from IGF-1Ea, but its role in muscle inflammation has not yet been characterized. In this study, we hypothesized that MGF expression in muscle injury modulates muscle inflammation. We first investigated changes of transcription and expression of MGF in response to skeletal muscle injury induced by cardiotoxin (CTX) *in vivo*. At 1–2 days post-injury, *Mgf* expression was significantly upregulated and positively correlated with that of inflammatory cytokines. Immunostaining revealed that infiltration of neutrophils and macrophages coincided with *Mgf* upregulation. Furthermore, infiltrating neutrophils and macrophages expressed *Mgf*, suggesting their contribution to MGF upregulation in muscle injury. Macrophages seem to be the predominant source of MGF in muscle injury, whereas neutrophil depletion did not affect muscle *Mgf* expression. Given the association of MGF and macrophages, we then studied whether MGF could affect macrophage infiltration and polarization. To test this, we overexpressed MGF in CTX-injured muscles and evaluated inflammatory marker expression, macrophage populations, and muscle regeneration outcomes. MGF overexpression delayed the resolution of macrophages, particularly the pro-inflammatory phenotype. This coincided with upregulation of inflammatory markers. Annexin V-based flow cytometry revealed that MGF overexpression likely delays macrophage resolution by limiting macrophage apoptosis. Although MGF overexpression did not obviously affect muscle regeneration outcomes, the findings are novel and provide insights on the physiological roles of MGF in muscle regeneration.

## Introduction

Skeletal muscle repair following injury involves a well-orchestrated series of events. The inflammatory response plays an essential role of the repair and regeneration processes. During this process, myeloid cells such as neutrophils and macrophages remove debris and secrete soluble factors to facilitate healing (Tidball and Rinaldi, 2012; Tidball, 2017). Numerous studies investigated the role of inflammatory cytokines, chemokines, and growth factors in mediating muscle inflammatory response (Pelosi et al., 2007; Tidball and Welc, 2015; Tonkin et al., 2015; Tidball, 2017). One of these factors, insulin-like growth factor 1 (IGF-1), is important for both resolving muscle inflammation and promoting myogenesis.

Due to alternative splicing, murine IGF-1 exists in two distinct isoforms, IGF-1Ea (GenBank: AY878192) and mechano-growth factor (MGF or IGF-1Eb; GenBank: AY878193). There are some similarities between the two isoforms but also significant differences (Dai et al., 2010). Both isoforms share the same mature IGF-1 peptide sequence containing exons 3 and 4, yet IGF-1Ea and MGF are characterized by their unique E-domain sequences composed of exons 4–6 and exons 4–5–6, respectively. The addition of exon 5 produces a frame shift in MGF E-domain (Brisson and Barton, 2012; Hede et al., 2012). In a microarray study, Barton et al. (2010) compared the transcriptomes of muscles overexpressing either IGF-1Ea or MGF. Of the 216 total modified genes, 30 were uniquely modified by MGF and 66 genes by IGF-1Ea. Thus, these two isoforms exhibit unique effects on modifying muscle gene expression. In transgenic mice model, it has been shown that MGF facilitates IGF-1 bioavailability by sequestrating IGF-1 in the extracellular matrix via its highly positively charged E-domain (Gallagher, 2001; Hede et al., 2012). Furthermore, the two IGF-1 isoforms show differential actions in the regulation of muscle growth. For instance, it has been shown *in vivo* that overexpression of MGF exerts a more potent hypertrophic effect than IGF-1Ea (Brisson et al., 2014).

Previous studies have shown that IGF-1 modulates muscle inflammation and regeneration and this has been primarily attributed to IGF-1Ea. Specifically, IGF-1Ea promotes macrophage polarization toward anti-inflammatory phenotype and downregulates the expression of inflammatory cytokines and chemokines (Pelosi et al., 2007; Tonkin et al., 2015). These effects were not attributed to MGF, in part because injured muscles from systemic MGF knockout mice do not display histological abnormalities (Tonkin et al., 2015). However, the potential compensatory mechanisms linked to the systemic loss of MGF could result in absence of phenotypic changes in muscle regeneration. Numerous studies have reported upregulation of MGF in response to muscle injury as early as 2–24 h post-injury (Hameed et al., 2008; McKay et al., 2008; Philippou et al., 2009), a time at which the damaged tissues were infiltrated by inflammatory cells (Tidball and Welc, 2015). Furthermore, the upregulation of MGF preceded that of IGF-1Ea (McKay et al., 2008; Philippou et al., 2009). Thus the physiological role of MGF appears to be involved in the muscle inflammatory process, distinct from IGF-1Ea.

Resident skeletal muscle cells, including myofibers, satellite cells, endothelial cells, and fibroblasts all contribute to endogenous IGF-1 expression (Christov et al., 2007; Ceafalan et al., 2014; Tonkin et al., 2015). Following muscle injury, however, the predominant source of IGF-1 upregulation is infiltrating myeloid cells (Tonkin et al., 2015). Yet the identity of myeloid progeny contributing to MGF upregulation in muscle injury is not known. Given of the differential infiltration dynamics of neutrophils and macrophages in muscle injury (Tidball, 2017), the expression and functions of MGF may vary depending on the predominant myeloid progeny within the injured muscle at a given time.

Considering the potent modulatory effects of IGF-1 in muscle inflammation, and the unique sequence identity and biological activity of MGF (Barton, 2006; Barton et al., 2010; Brisson and Barton, 2012), it seems plausible that MGF plays immunomodulatory functions in muscle regeneration distinct from IGF-1Ea. However, relatively few studies have characterized the role of MGF in the context of inflammation after muscle injury. In the present study, we hypothesize that MGF modulates the inflammatory response in muscle injury. To test this, we injected cardiotoxin (CTX) into the tibialis anterior (TA) muscle as the model of injury. To determine the contribution of myeloid cells to MGF upregulation, we analyzed the expression of MGF in neutrophils and macrophages isolated from the injured muscles. We further overexpressed full-length MGF in the injured TA muscle and analyzed the expression of inflammatory cytokines, profile of infiltrating macrophages, and muscle regeneration outcomes at various timepoints after injury.

## Materials and Methods

This study was carried out in accordance with the recommendations in the Guide for the Care and Use of Laboratory Animals of the National Institutes of Health. The protocol was approved by the Animal Subjects Ethics Sub-Committee of The Hong Kong Polytechnic University (ASESC no: 16-17/82).

### Animals

Adult male mice (BALB/c; 10–14 weeks old; body weight 24.47 ± 1.12 g) were obtained from Centralized Animal Facilities at The Hong Kong Polytechnic University. Mice were housed in cages and maintained under a 12:12 h dark–light cycle with controlled temperature (19–26°C) and humidity (50–60%). Animals were allowed free-cage movement and *ad libitum* access to food and water during holding and after experimental procedures.

### Muscle Injury Model

Intramuscular CTX injection is a widely accepted muscle injury model that induces characteristic myofiber necrosis followed by inflammation and muscle regeneration (Musarò et al., 2001; Wang et al., 2014; Hardy et al., 2016). To induce muscle injury, animals were first anesthetized with isoflurane (1.5–2%), and then received a single intramuscular injection of filter-sterilized CTX solution (10 μM in 0.9% m/v saline, 1 μl/g body weight; Sigma-Aldrich, United States) at the TA mid-belly. The injured muscle was harvested at 0 (baseline control), 2, 8 h, 1, 2, 4, and 7 days post-injury for further analysis.

### Neutrophil Depletion Animal Model

Antibody against mouse Ly6G (Clone 1A8) has been previously reported to successfully deplete circulating neutrophils in skeletal muscle injury (Daley et al., 2008; Kawanishi et al., 2016). In this study, anti-Ly6G antibody (500 μg in 200 μl of 0.9% saline; Bio X Cell, United States) were intraperitoneally injected 2 days before CTX injection to inhibit neutrophil infiltration into injured muscles. Animals injected with isotype control antibody (Clone 2A3; Bio X Cell, United States) served as negative controls.

### Construction of MGF Expression Plasmid

Total RNA from untreated TA muscles was extracted and isolated by SV Total RNA Isolation System (Promega, United States). cDNA synthesis was performed using the GoScript Reverse Transcription System (Promega, United States). Construction of MGF expression plasmid was carried out as previously described (Xu et al., 2008). In brief, the coding sequencing of MGF was amplified by PCR and then cloned into the *Bam*HI and *Not*I restriction sites of the expression vector pcDNA 3.1+ (Thermo Fisher Scientific, United States). Specifically, the cloned sequence encodes the class I signal peptide, mature IGF-1 peptide, followed by the MGF E-peptide. Due to the lack of commercially available and validated antibodies against MGF, a hexahistidine epitope tag (6-HIS) was fused to the C-terminal end of MGF E-peptide in the recombinant plasmid. After the DNA sequence was confirmed, the expression plasmid, pMGF, was purified using the EndoFree Plasmid Maxi Kit (QIAGEN, United States) for transfection assays.

### *In Vivo* MGF Transfection by Electroporation

**FIGURE 1:**
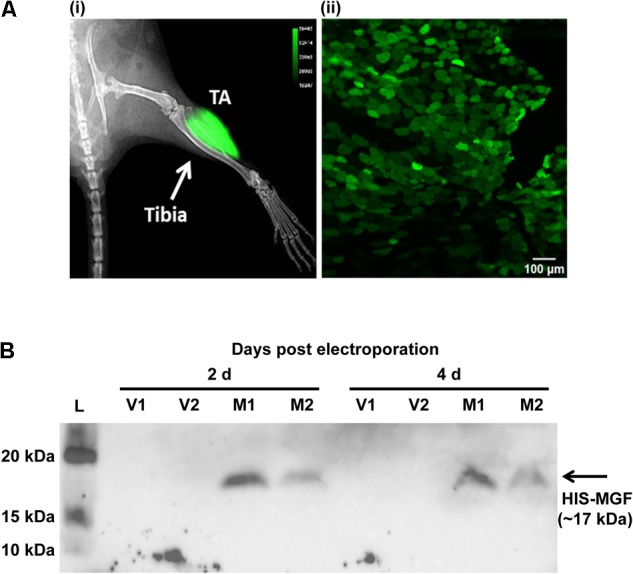
*In vivo* transfection by electroporation and overexpression of mechano-growth factor (MGF) peptide in tibialis anterior (TA) muscle. **(A)** *In vivo* imaging by Xtreme II (Bruker, United States) and histology of pEGFP-N1 electroporated TA muscle. **(i)** The overlaid image of radiograph and fluorescence image of mouse lower limb showing intense green fluorescence protein (GFP) signal focally at the TA muscle. **(ii)** The fluorescence image of the cross-section of the TA muscle showing GFP-expressing myofibers within muscle section. Scale = 100 μm. Intense green florescence protein (GFP) signal was detected focally on the lateral side of the tibia. The green polygonal structures of the TA muscle cryosections represented the myofibers expressing GFP. **(B)** SDS-PAGE and Western blot of muscles electroporated with pMGF. Samples were harvested at 2 and 4 days post-electroporation. L, protein ladder; M, samples electroporated with pMGF; V, samples electroporated with empty vector.

Expression plasmid pMGF was *in vivo* transfected using electroporation. Briefly, animals were first anesthetized, and then the TA muscle was injected with 20 μl of filter-sterilized bovine hyaluronidase (0.4 U/μl in 0.9% m/v saline; Sigma-Aldrich, United States). Two hours later, 25 μg of filter-sterilized pMGF plasmid (1 μg/μl in 0.45% m/v saline) was injected. The use of both hyaluronidase (McMahon et al., 2001) and 0.45% m/v saline (Lee et al., 2002) maximizes electroporation efficiency. Immediately after pMGF injection, platinum tweezertrodes (5 mm diameter, BTX, United States) were placed longitudinally relative to the TA muscle, and electroporation was performed by applying 10 square-wave pulses (ECM830, BTX, United States) of 20 ms and 175 V/cm at a frequency of 1 Hz (McMahon et al., 2001). The contralateral TA muscle was electroporated with mock vector (pcDNA 3.1) in the same manner and served as a negative control. Specific transfection of TA muscle by electroporation and the expression of 6-HIS tagged MGF peptide were demonstrated by *in vivo* imaging and Western blotting, respectively (**Figure 1**).

### *In Vitro* MGF Overexpression in Culture Cell Line

To study the effects of MGF overexpression in muscle cells, C2C12 murine myoblast cells were cultured and differentiated in Dulbecco’s modified Eagle’s medium as previously described (Cheung et al., 2011). In 12-well plates, myoblasts and myotubes were transfected with pMGF or mock vector (2 μg/ml DNA per well) using Lipofectamine 2000 (Thermo Fisher Scientific, United States). Cells were harvested at 24 and 48 h post-transfection for transcript evaluation.

### Cell Isolation and Flow Cytometry

Muscle disaggregation and myeloid cell isolation were performed as described in Tonkin et al. (2015). To isolate white blood cells, blood was collected by cardiac puncture. Red blood cells were lysed with two volumes of working BD Pharm Lyse (BD Biosciences, United States).

Cells from muscle and blood were reconstituted into 1 × 10^6^ cells/100 μl of phosphate-buffered saline (PBS) containing 1% fetal bovine serum and anti-mouse CD16/CD32 antibody (1 μg/1 × 10^6^ cells; BD Biosciences, United States). The cells were then incubated with the desired antibodies, including: CD11b-BB515 (Clone M1/70, 0.5 μg/1 × 10^6^ cells; BD Biosciences, United States), Ly6C-PE (Clone HK 1.4, 1 μg/1 × 10^6^ cells; BioLegend, United States), Ly6G-PE-Cy7 (Clone 1A8, 1 μg/1 × 10^6^ cells; BioLegend, United States), CD206 (Clone C068C2, 1 μg/1 × 10^6^ cells; BioLegend, United States), and F4/80-AF647 (Clone T45-2342, 2 μg/1 × 10^6^ cells; BD Biosciences, United States). Annexin V-PE (5 μl/1 × 10^6^ cells; BioLegend, United States) was used to stain apoptotic macrophage. 7-AAD (10 μl/1 × 10^6^ cells; BD Biosciences, United States) served as the viability dye. Flow cytometry was performed using Accuri C6 Flow Cytometer (BD Biosciences, United States), and data analysis was performed using Accuri C6 software (BD Biosciences, United States). To sort cells for real-time quantitative PCR, fluorescence activated cell sorting experiments were conducted using BD FACSAria III Cell Sorter controlled by BD FACSDiva software (BD Biosciences, United States).

### Real-Time Quantitative PCR

Total RNA from dissected muscle was isolated by the SV Total RNA Isolation System (Promega, United States). For the sorted cells, RNA was extracted by the ReliaPrep RNA Cell Miniprep System (Promega, United States). For the C2C12 cell cultures, PureLink RNA Mini Kit was applied. The concentration and purity of extracted RNA was determined by measuring absorbance at 260 and 280 nm.

From each sample, 1 μg of RNA was converted into cDNA by GoScript Reverse Transcription System (Promega, United States). The expression of transcripts against the mouse genome was studied by Taqman assays (Supplementary Table 1; Thermo Fisher Scientific, United States) using the CFX Connect Real-time PCR Detection System (Bio-Rad, United States). The selection of the internal control genes was determined by NormFinder (Andersen et al., 2004). Fold change in gene expression between treatment groups was calculated using the 2^-ΔΔCt^ relative quantification method.

### Western Blotting

Total protein was extracted from muscles as previously described (Cheung et al., 2011; Xia et al., 2016). The protein samples were analyzed on a 4–15% gradient gel followed by Western transfer to a 0.2-μm nitrocellulose membrane (PerkinElmer Life Sciences, United States). Mouse THE^TM^ His-tag monoclonal antibody (GenScript, United States) at 0.2 μg/ml final concentration was used to probe for the expression of HIS-tagged MGF. After incubating with the secondary antibody (horseradish peroxidase-conjugated anti-mouse IgG, 1:1000), the immunoblotting signal was detected by ECL Chemiluminescent Kit and ChemiDoc Imaging System (Bio-Rad, United States).

### Histology and Immunohistochemistry

Isolated TA muscles were snap-frozen in liquefied nitrogen-chilled isopentane (Sigma-Aldrich, United States), cryoembedded and cryosectioned at 7-μm thickness. All sections were fixed with paraformaldehyde solution (4% in PBS) for 10 min, followed by either hematoxylin and eosin (H&E) staining or immunohistochemistry, as previously described (Zhang et al., 2014; Xia et al., 2016).

To evaluate muscle histology, images covering entire H&E-stained muscle sections were captured for analyses of centrally nucleated myofibers and myofiber cross-sectional area. For immunohistochemistry, primary antibodies, including rat anti-mouse Ly6G (Clone 1A8; 5 μg/ml; BD Biosciences, United States) and rat anti-mouse F4/80 (CI:A3-1; 10 μg/ml; Bio-Rad, United States); and secondary antibodies, including Alexa Fluor 488- or 568-conjugated goat anti-rat secondary antibodies (5 μg/ml; Thermo Fisher Scientific, United States) were used in the present study. The injury loci were identified and the fluorescent images were captured for qualitative analyses. All images were captured using an Eclipse 80i microscope (Nikon, Japan) equipped with a SPOT-camera (SPOT Imaging, United States) and analyzed with ImageJ image analysis software (National Institutes of Health, United States).

### Statistical Analyses

Data were analyzed using SPSS24 software (IBM, United States). Pearson correlation was performed to evaluate the association of MGF with inflammatory cytokines. Comparisons between macrophages isolated from pMGF + CTX and vector + CTX muscles were analyzed by Student’s *t*-test. Comparisons of gene expression amongst (i) timepoints post-CTX-induced injury and (ii) myeloid cells isolated from injured muscle were analyzed by one-way ANOVA. Two-way ANOVA was used to analyze the main effect of treatments between (i) pMGF vs. vector groups and (ii) pMGF + CTX vs. vector + CTX groups as well as the main effect of time (amongst evaluation timepoints). *Post hoc* Bonferroni tests were performed when significance was detected by one-way and two-way ANOVA tests. *P* < 0.05 was considered statistically significant. All values are expressed as the mean ± SEM.

## Results

### MGF Is Upregulated in Muscle Injury and Is Associated With Inflammatory Markers

**FIGURE 2:**
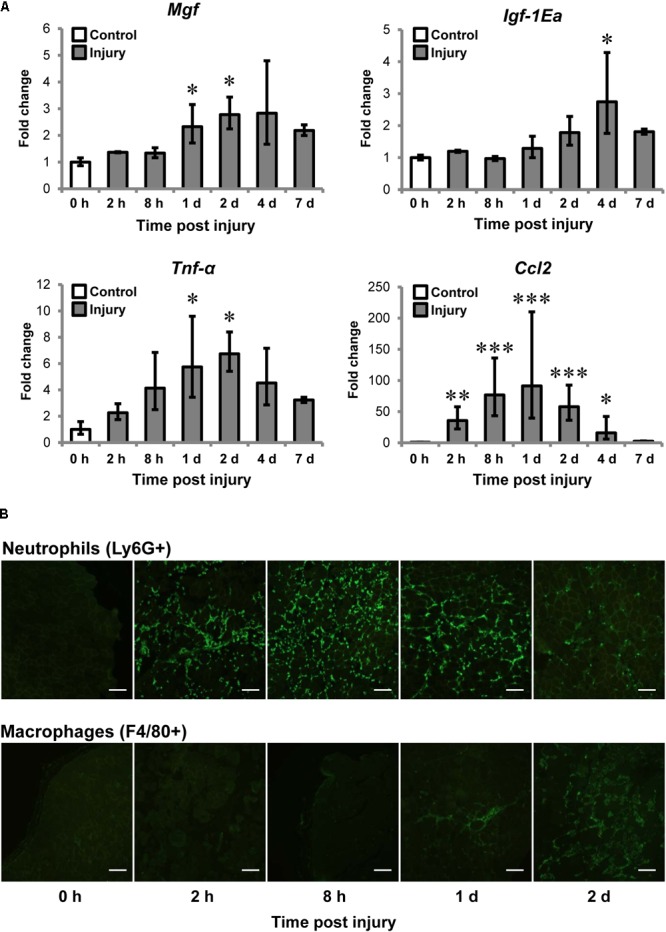
Upregulation of MGF in muscle injury and the association with inflammatory markers. Cardiotoxin (CTX)-injured muscles were harvested at 0, 2, 8 h, 1, 2, 4, and 7 days post-injury. Muscles at 0 h were not injected with CTX, serving as the uninjured control. **(A)** Expression of *Mgf Igf-1Ea*, and inflammatory cytokines in muscle injury (*n* = 4–6/timepoint). *Gapdh* expression served as the internal control. The expression level is relative to that at 0 h. Significant differences from 0 h: ^∗^*P* < 0.05, ^∗∗^*P* < 0.01, and ^∗∗∗^*P* < 0.001. Statistics were analyzed by one-way ANOVA and followed by Bonferroni test. Values represent mean ± SEM. **(B)** Infiltration of neutrophils and macrophages in muscle injury. Immunopositive staining (green) of Ly6G^+^ and F4/80^+^ cells represent neutrophils and macrophages, respectively, on CTX-injured muscle sections. Consecutives sections were used and the same injured loci were evaluated. Scale = 100 μm.

We first examined MGF expression and muscle inflammation post-CTX-injury. The injured muscle was harvested at 0 (baseline control), 2, 8 h, 1, 2, 4, and 7 days post-injury for gene expression analysis (*n* = 4–6/timepoint) and immunohistochemistry of neutrophils and macrophages. *Mgf* expression was significantly increased at 1 and 2 days post-injury (both *P* < 0.05; **Figure 2A**) and preceded that of IGF-1Ea. *Igf-1Ea* expression was not upregulated until 4 days post-injury.

It is known that expression of pro-inflammatory cytokines, such as tumor necrosis factor-α (TNF-α) and C–C motif ligand 2 (CCL2) elevated after injury (Tidball et al., 2014; Tidball, 2017). For this, we examined *Tnf-α* and *Ccl2* expression and they were significantly upregulated at 1 and 2 days compared to control. Pearson correlation tests confirmed that *Mgf* expression correlated with expression of *Tnf-α* (*R* = 0.72; *P* < 0.001) and *Ccl2* (*R* = 0.52; *P* < 0.01).

Given *Mgf* was upregulated at the same time course as the pro-inflammatory cytokines, we next characterized the infiltration of myeloid cells, predominantly neutrophils and macrophages (Tidball, 2017), for the first 2 days post-injury. Neutrophil infiltration (Ly6G^+^ cells) began as early as 2 h post-injury, then peak at 8 h and began to resolve by 2 days post-injury; whereas macrophages (F4/80^+^ cells) were observable at 1 day and dominated the injury site at 2 days (**Figure 2B**). This observation suggests that the MGF upregulation may be associated with the inflammatory response, particularly with the infiltration of macrophages.

### Macrophages Are the Major Myeloid Cells Contributing to MGF Upregulation in Muscle Injury

Myeloid cells are known to be a predominant source of IGF-1 upregulation in muscle injury (Tonkin et al., 2015). To determine which of these cell types expressed MGF in muscle injury, we used cell sorting to isolate neutrophils and macrophages from muscles 36 h post-injury, and then examined *Mgf* expression in these isolated myeloid cells. The gating information for myeloid cell sorting is shown in Supplementary Figure 1.

**FIGURE 3:**
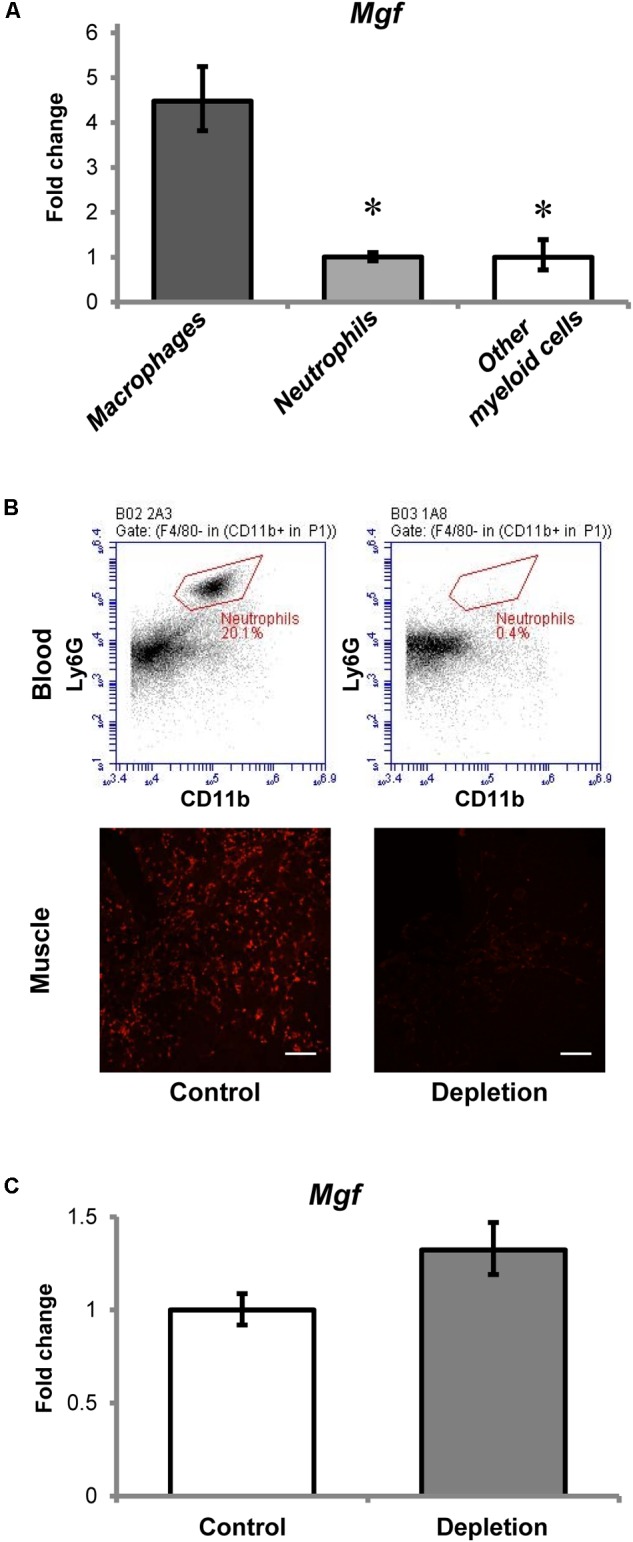
Contribution of myeloid cells to MGF upregulation in muscle injury. **(A)** Expression of *Mgf* in myeloid cells isolated from muscles at 36 h post-injury (*n* = 4–6/myeloid cell type). *Gapdh* expression served as the internal control. The expression level is relative to that of other myeloid cells. Significant differences from macrophages: ^∗^*P* < 0.05. Statistics were analyzed by one-way ANOVA and followed by Bonferroni test. Values represent mean ± SEM. **(B)** Effect of anti-Ly6G antibody administration on circulating neutrophils and neutrophil infiltration into CTX-injured muscle at 1 day post-injury. This is compared with control treated with isotype control antibody. The upper panel demonstrates the flow cytometric analysis of neutrophils (CD11b^+^ F4/80^-^ Ly6G^+^ cells) in blood, while the lower panel indicates the Ly6G immunopositive staining (red) representing neutrophils in muscle injury. Scale = 100 μm. **(C)** Expression of *Mgf* in neutrophil-depleted CTX-injured muscles at 1 day post-injury (*n* = 7/treatment). *Gapdh* expression served as the internal control. This is compared with control treated with isotype control antibody. Values represent mean ± SEM.

All myeloid cells isolated from the injured muscles expressed *Mgf* (**Figure 3A**), but *Mgf* expression was higher in macrophages (7AAD^-^ Ly6G^-^ CD11b^+^ Ly6C^+^ F4/80^+^ cells) than in neutrophils (7AAD^-^ CD11b^+^ F4/80^-^ Ly6C^+^ Ly6G^+^ cells) and other myeloid cells (7AAD^-^ Ly6G^-^ CD11b^+^ Ly6C^+^ F4/80^-^ cells). Although neutrophils expressed *Mgf*, the timing of *Mgf* upregulation and neutrophil infiltration did not coincide. Specifically, infiltrated neutrophils were present in the injured muscles as early as 2 h post-injury, while transcript of *Mgf* was not found until 1 day post-injury.

To evaluate further the role of neutrophils to *Mgf* expression in muscle injury, we quantified *Mgf* expression 1 day post-injury in neutrophil-depleted animals (*n* = 7/treatment/timepoint). This timepoint was chosen because it coincided with the peak of neutrophil infiltration and the emergence of significant *Mgf* upregulation. Flow cytometric analysis (see Supplementary Figure 2 for gating information) confirmed that in this neutrophil depletion model (**Figure 3B**), circulating neutrophils were depleted and the concentration of infiltrating neutrophils in injured muscles was reduced as described previously (Kawanishi et al., 2016). Of importance, *Mgf* expression was not affected by neutrophil depletion (**Figure 3C**), suggesting that neutrophils were not necessary for MGF upregulation in muscle injury. This corresponds with the results of our cell sorting experiments that macrophages expressed a higher level of *Mgf* relative to neutrophils. Considering the concurrent *Mgf* expression and macrophage infiltration, it is possible that MGF may modulate macrophage activity and muscle inflammation. Thus, we performed experiments to overexpress MGF in the TA muscle then followed by CTX-induced injury to investigate the potential effects of MGF on infiltrated macrophages.

### Increased Cytokine Expression in Muscle Injury Upon MGF Overexpression Is Not Due to Enhanced Macrophage Transcriptional Activity

**FIGURE 4:**
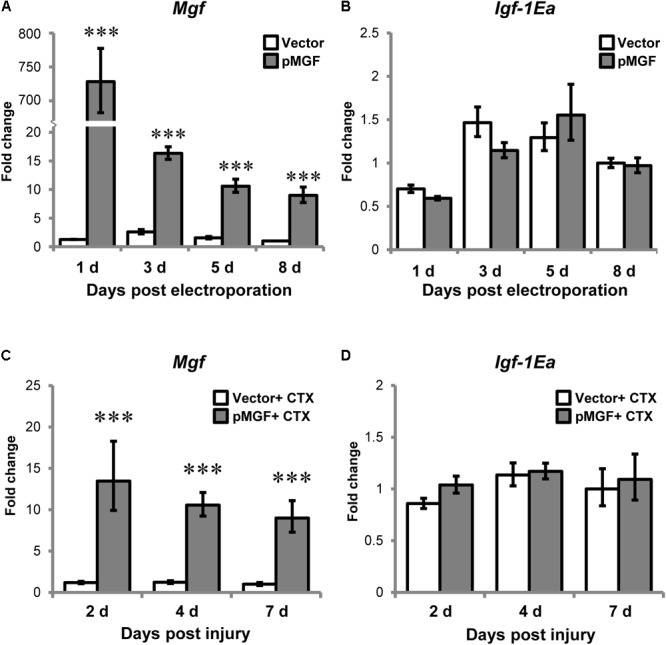
Specific overexpression of MGF in pMGF-electroporated muscles. The expression of **(A)** *Mgf* and **(B)** *Igf-1Ea* in pMGF-electroporated muscles harvested from 1 to 8 days post-electroporation (*n* = 5/treatment/timepoint). This is relative to 8 days vector. Significant difference between vector and pMGF: ^∗∗∗^*P* < 0.001. The expression of **(C)** *Mgf* and **(D)** *Igf-1Ea* in pMGF-electroporated CTX-injured muscles harvested from 2 to 7 days post-injury (*n* = 6/treatment/timepoint). This is relative to 7 days vector + CTX. Significant difference between vector + CTX and pMGF + CTX: ^∗∗∗^*P* < 0.001. The geometric mean of *Gapdh, 18S* rRNA, and *Rsp20* expression served as the internal control. Statistics were analyzed by two-way ANOVA and followed by Bonferroni test. Values represent mean ± SEM.

Electroporation of pMGF plasmid in intact muscle sustainably increased *Mgf* expression relative to vector treatment throughout an 8-day period, which is essential for the observation of the time course of inflammatory events (*P* < 0.001; **Figure 4A**). However, overexpression of MGF did not induce any changes in *Igf-1Ea* expression (**Figure 4B**).

After verifying our overexpression model, muscles were electroporated with either pMGF or mock vector and injected with CTX solution the next day. CTX injury did not abolish the sustainable MGF overexpression (**Figure 4C**). The expression of *Igf-1Ea* in the context of muscle injury was also not affected by MGF overexpression (**Figure 4D**). These suggest that findings observed in our experiments can be attributed to the overexpression of MGF.

**FIGURE 5:**
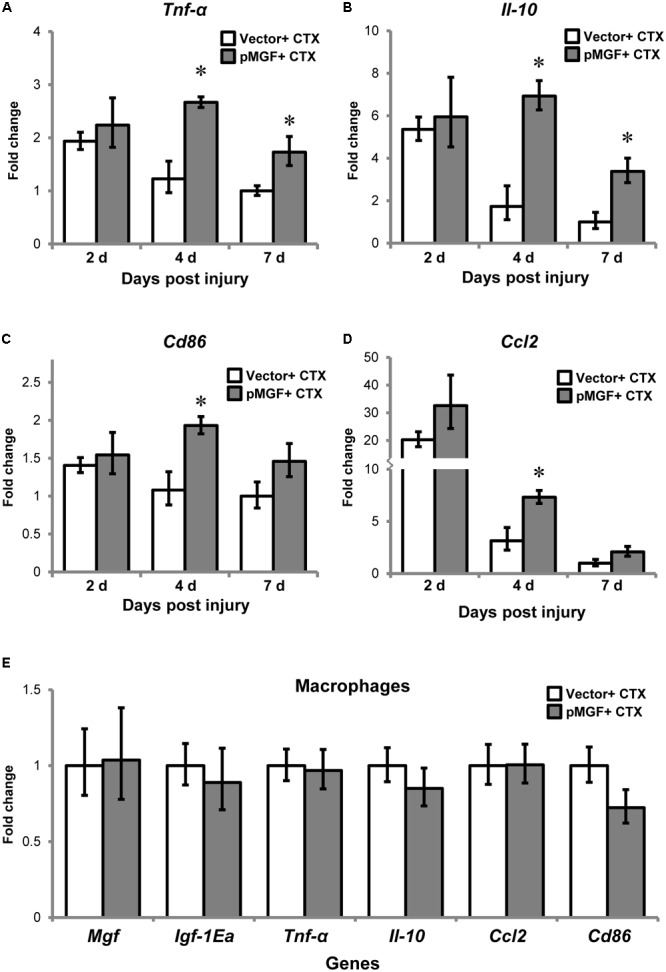
Upregulation of inflammatory genes by MGF overexpression in muscle injury without altering macrophage transcription. The expression of genes related to muscle inflammation, including **(A)** *Tnf-α*, **(B)** *Il-10*, **(C)** *Cd86*, and **(D)** *Ccl2*. Samples were collected from 2 to 7 days post-injury (*n* = 6/treatment/timepoint). The geometric mean of *Gapdh, 18S* rRNA, and *Rsp20* expression served as the internal control. The expression level is relative to that of 7 days vector + CTX. Student’s *t*-test was used to compare between vector + CTX and pMGF + CTX groups. ^∗^*P* < 0.05. **(E)** Expression of IGF-1 isoforms and inflammatory markers (*Tnf-α, Il-10, Cd86*, and *Ccl2*) in macrophages. Macrophages were isolated at 4 days post-injury (*n* = 6/treatment). The geometric mean of *Gapdh* and *18S* rRNA expression served as the internal control. This is relative to vector + CTX. Values represent mean ± SEM.

One prominent function of macrophages is mediating muscle inflammation by expressing and secreting various inflammatory cytokines and chemokines after injury (Tidball, 2017). We therefore evaluated and compared inflammatory cytokines and macrophage markers in MGF-overexpressing CTX-injured muscles relative to vector-electroporated CTX-injured muscles (hereafter, pMGF + CTX and vector + CTX, respectively) at 2, 4, and 7 days post-injury (*n* = 6/treatment/timepoint). As shown in **Figures 5A–D**, the expression of inflammatory markers in vector + CTX injury treatment displayed a reducing trend from 2 to 7 days post-injury. Comparatively, overexpression of MGF followed the same reducing trend but demonstrated a significantly higher expression level than vector control. The pMGF + CTX muscles expressed significantly more cytokines *Tnf-α* and interleukin-10 (*Il-10*), pro-inflammatory macrophage marker *Cd86* and chemokine *Ccl2* (*P* < 0.05 for all) than vector + CTX ones at 4 days. The upregulation for *Tnf-α* and *Il-10* upon pMGF + CTX treatment remained significant at 7 days. Expression of interleukin-6 (*Il-6*) and the anti-inflammatory macrophage marker, *Cd206*, also demonstrated a significant major effect of MGF overexpression in a two-way ANOVA (pMGF + CTX > vector + CTX; both *P* < 0.05), although there were no significant differences between groups in *post hoc* tests. In summary, MGF overexpression delayed the downregulation of inflammatory markers in muscle injury, with the most significant differences from controls at 4 days post-injury.

To determine if MGF overexpression modulates gene expression in inflammatory cells, we isolated macrophages from muscles by sorting at 4 days post-injury (*n* = 3/cell/treatment). The results showed that none of the above inflammatory markers were upregulated in the isolated macrophages (**Figure 5E**). Thus, the changes in transcript level observed in 4 days post-injury was not due to modulation in macrophage transcriptional activity.

### Muscle Cells Do Not Upregulate Inflammatory Cytokines in Response to MGF Overexpression

**FIGURE 6:**
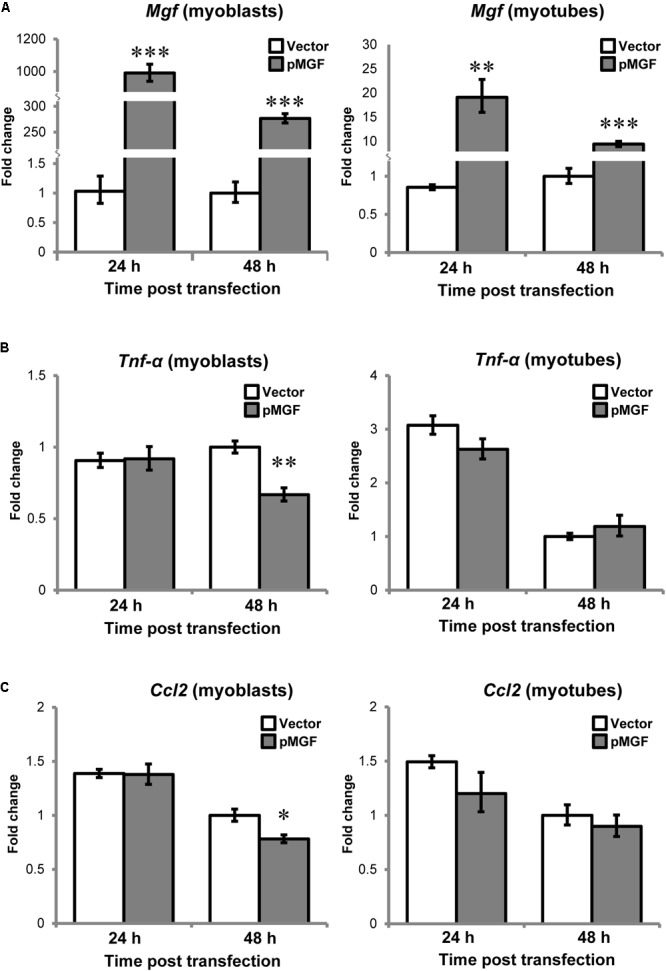
No observable change in inflammatory cytokine expression in MGF-overexpressing C2C12 myogenic cells *in vitro*. The expression of **(A)** *Mgf*, **(B)** *Tnf-α*, and **(C)** *Ccl2* in myoblast and myotube cultures harvested at 24 and 48 h post-transfection (*n* = 3/cell/treatment/timepoint). *Gapdh* expression served as the internal control. The expression level is relative to 48 h vector control. Significant difference between vector and pMGF: ^∗^*P* < 0.05, ^∗∗^*P* < 0.01, and ^∗∗∗^*P* < 0.001. Statistics were analyzed by two-way ANOVA and followed by Bonferroni test. Values represent mean ± SEM.

Since muscle cells also express cytokines or myokines, such as TNF-α and CCL2 in response to injury (Collins and Grounds, 2001; Peake et al., 2015), we want to assess if there is potential contribution from muscle cells to the upregulation of cytokines at tissue level. C2C12 myoblast and myotube cells following MGF overexpression for either 24 or 48 h (*n* = 3/cell/treatment/timepoint; **Figure 6**) were harvested. In both myoblast and myotube culture, *Tnf-α* and *Ccl2* were not upregulated in response to MGF overexpression. Expression of *Il-10* was not detected in muscle cells, similar to the previous study (Peake et al., 2015). Thus, the upregulation of inflammatory cytokines at tissue level was unlikely due to modulation in muscle cell transcriptional activity.

### MGF Overexpression in Muscle Injury Delays the Resolution of Pro-inflammatory Macrophages

**FIGURE 7:**
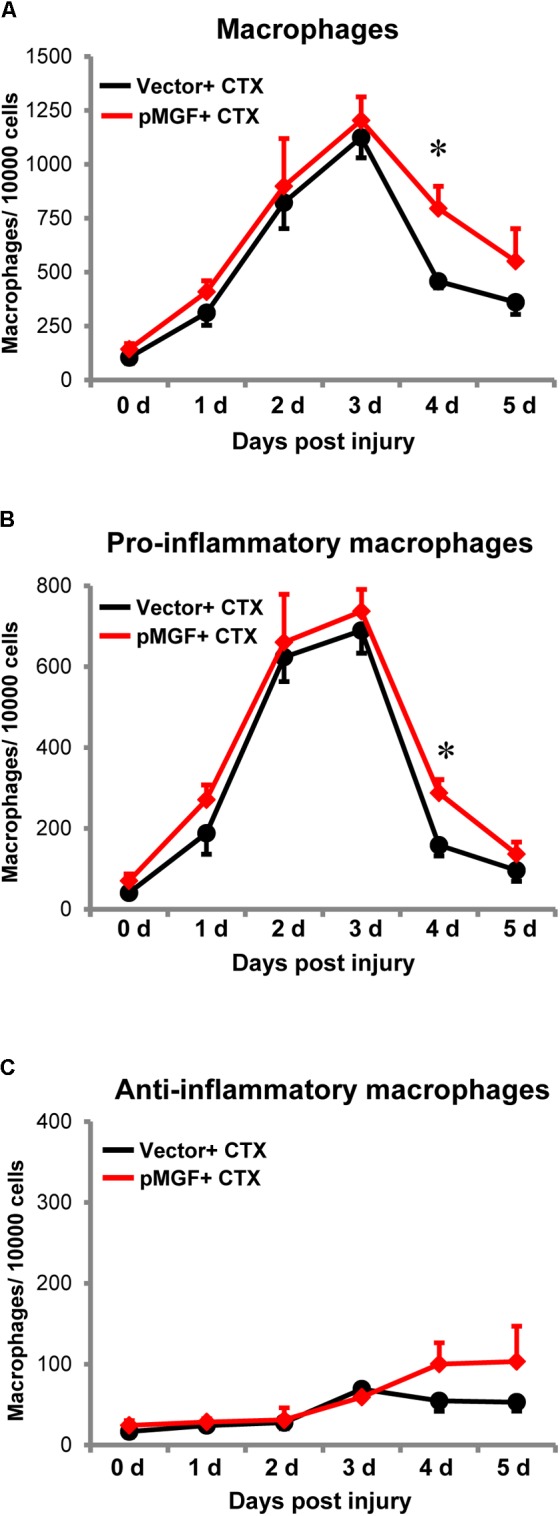
Delayed resolution of macrophages by MGF overexpression in muscle injury. Samples were harvested daily from 0 to 5 days post-injury (*n* = 4–6/treatment/timepoint). **(A)** Total macrophages (CD11b^+^ F4/80^+^), **(B)** pro-inflammatory macrophages (CD11b^+^ F4/80^+^ Ly6C^+^ CD206^-^), and **(C)** anti-inflammatory macrophages (CD11b^+^ F4/80^+^ Ly6C^-^ CD206^+^) were evaluated. The black line represents vector + CTX whereas the red line represents pMGF + CTX. Significant difference between vector + CTX and pMGF + CTX: ^∗^*P* < 0.05. Statistics were analyzed by two-way ANOVA and followed by Bonferroni test. Values represent mean ± SEM.

Increased expression of inflammatory genes in muscle injury might also result from changes in macrophage accumulation and polarization (Tidball, 2017). Therefore, we investigated the infiltration and resolution of macrophages in MGF-overexpressing CTX-injured muscles. Using flow cytometry, we evaluated macrophages and the subpopulations from 0 day baseline until 5 days post-injury (*n* = 4–6/treatment/timepoint; **Figure 7**). The number of total macrophages (CD11b^+^ F4/80^+^), pro-inflammatory macrophages (CD11b^+^ F4/80^+^ Ly6C^+^ CD206^-^), and anti-inflammatory macrophages (CD11b^+^ F4/80^+^ Ly6C^-^ CD206^+^) were evaluated (see Supplementary Figure 3 for gating strategy).

An increase in macrophages, predominantly the pro-inflammatory population occurred from 0 to 3 days post-injury, indicating macrophage infiltration. The number returned to baseline levels at 5 days, indicating resolution of this subpopulation. During this resolution phase at 4 days post-injury, pMGF + CTX treatment significantly increased both the number of total macrophages and specifically pro-inflammatory macrophages relative to vector + CTX treatment (*P* < 0.05 for both). For the anti-inflammatory macrophages, the population increased during the evaluation time course (major effect of “days post-injury” from two-way ANOVA, *P* < 0.05). However, the emergence of anti-inflammatory macrophages was unaffected by MGF overexpression in this injury model. MGF overexpression appears to influence the resolution of pro-inflammatory macrophages in muscle injury.

### MGF Overexpression in Muscle Injury Attenuates Macrophage Apoptosis

**FIGURE 8:**
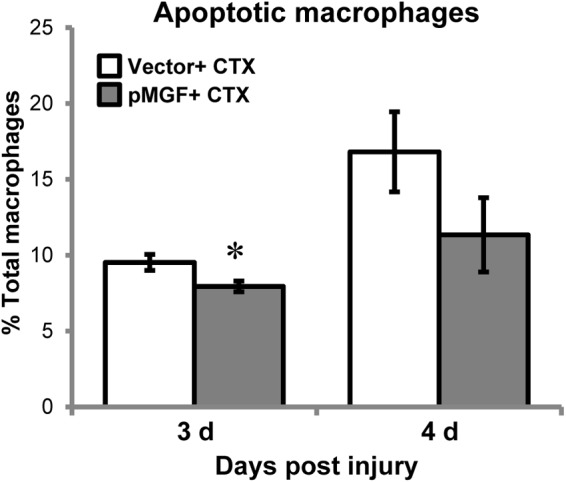
Macrophage apoptosis in MGF-overexpressing CTX-injured muscles. Samples were harvested at 3 and 4 days post-injury (*n* = 5/treatment/timepoint). Apoptotic macrophages (CD11b^+^ F4/80^+^ Annexin V^+^ 7-AAD^+^) were examined. Significant difference between vector + CTX and pMGF + CTX: ^∗^*P* < 0.05. Statistics were analyzed by two-way ANOVA and followed by Bonferroni test. Values represent mean ± SEM.

During inflammatory resolution, macrophages disappear primarily by undergoing apoptosis (Sciorati et al., 2016). This raised a question of whether the appeared delay of inflammatory resolution mediated by MGF overexpression could potentially be a result of alteration of macrophage apoptosis. We quantified apoptotic macrophage in MGF-overexpressing CTX-injured muscles by detecting Annexin V, an apoptotic marker, using flow cytometry. Macrophages were isolated at 3 and 4 days post-injury (*n* = 5/treatment/timepoint; **Figure 8**), during the time course of macrophage resolution. The gating strategy is shown in Supplementary Figure 4. The number of apoptotic macrophages (CD11b^+^ F4/80^+^ Annexin V^+^ 7-AAD^+^) increased from 3 to 4 days post-injury (major effect: *P* < 0.05), indicating progression of macrophage resolution. In MGF-overexpressing muscles, there were fewer apoptotic macrophages occurred at 3 days suggesting that MGF overexpression suppressed macrophage apoptosis at the onset of macrophage resolution.

### MGF Overexpression in Muscle Injury Does Not Seem to Affect Muscle Regeneration

**FIGURE 9:**
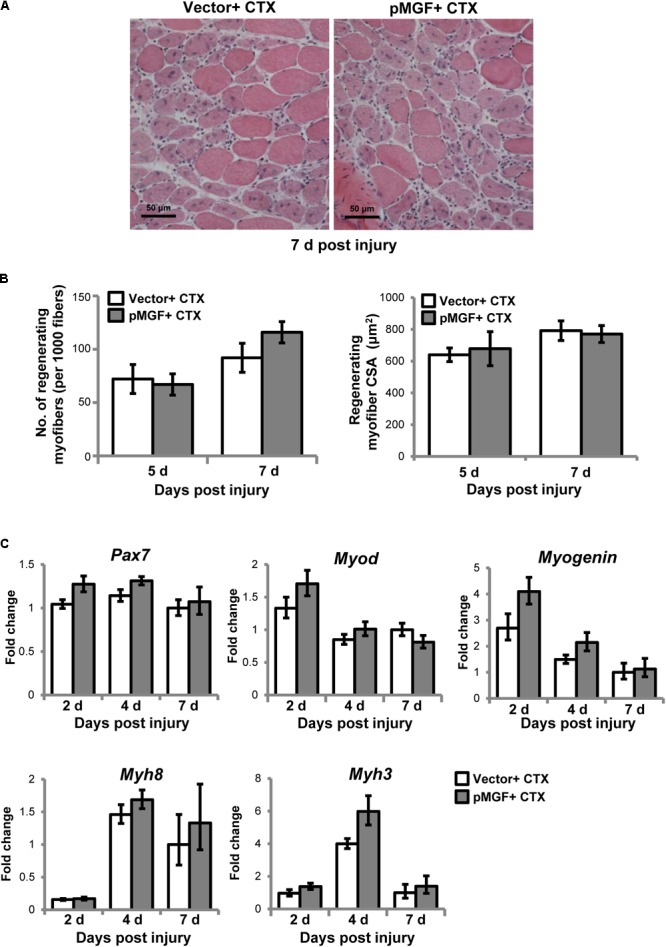
Muscle regeneration outcomes in MGF-overexpressing CTX-injured muscles. **(A)** Hematoxylin and eosin staining of muscles harvested at 7 days post-injury. Scale = 50 μm. **(B)** The number and cross-sectional area (CSA) of centrally nucleated myofibers (*n* = 4/treatment/timepoint). Values represent mean ± SEM. **(C)** Expression of genes related to muscle regeneration. Samples were collected from 2 to 7 days post-injury (*n* = 6/treatment/timepoint). The investigated genes included *Pax7, Myod, Myogenin, Myh3*, and *Myh8*. The geometric mean of *Gapdh, 18S* rRNA, and *Rsp20* expression served as the internal control. The expression level is relative to that of 7 days vector + CTX. Statistics were analyzed by two-way ANOVA and followed by Bonferroni test. Values represent mean ± SEM.

We next examined how the immunomodulatory effects of MGF translated to muscle regeneration outcomes. The MGF treatment did not affect the number nor the cross-sectional area of the centrally nucleated myofibers at 5 and 7 days post-injury (*n* = 4/treatment/timepoint; **Figures 9A,B**). We also examined the expression of genes associated with muscle regeneration, including paired-box protein 7 (*Pax7*), myogenic differentiation 1 (*Myod*), *Myogenin*, and embryonic (*Myh3*) and neonatal (*Myh8*) myosin heavy chain in MGF-overexpressing CTX-injured muscles (*n* = 6/treatment/timepoint; **Figure 9C**). MGF treatment did not influence the expression of any of these genes. Thus, under the present conditions, although MGF caused changes in inflammatory cytokines and resolution of macrophages, it did not seem to affect the regenerative response of injured muscle.

## Discussion

In this study, we observed how expression of MGF, a splice form of IGF-1, affected muscle inflammation in a CTX-induced model of muscle injury. MGF was upregulated after muscle injury, which coincided with inflammatory marker gene expression and infiltration of myeloid cells, suggesting an association with muscle inflammation. The predominant expression of MGF by infiltrating macrophages implies that MGF may modulate macrophage activities. Overexpressing MGF increased inflammatory cytokine gene expression and delayed the resolution of pro-inflammatory macrophages. Our data indicate that this likely resulted from increased macrophage accumulation in the injured muscle, as MGF overexpression inhibited macrophage apoptosis. Despite these changes in the muscle inflammatory response, MGF overexpression did not grossly affect muscle regeneration outcomes. Nevertheless, these data are novel in clarifying the role of MGF in muscle injury.

Previous investigations have relied on systemic injection of a MGF E-peptide to study the function of MGF. This MGF E-peptide analog corresponds to the last 24 amino acid residues at the C-terminal of E domain (see review: Zabłocka et al., 2012). In the present study, we further examine the role of MGF in muscle inflammatory response by overexpressing full-length MGF of murine origin containing the signal peptide, mature IGF-1 peptide and MGF E-peptide (Musarò et al., 2001; Barton et al., 2010; Brisson et al., 2014). We believe this to be important because of the following reasons: (i) mature IGF-1 peptide is biologically active and its activity can be modulated by MGF E-peptide by sequestration to the extracellular matrix (Hede et al., 2012). The inclusion of just the E domain may not fully reflect the activity of the entire MGF gene. (ii) MGF E-peptide from full-length MGF contains post-translational modification site (e.g., endopeptidase cleavage site) (Philippou et al., 2014). The MGF E-peptide analog, however, usually contains amino acid modification (e.g., L- to D-arginine) to protect the peptide from degradation. Such modification, though it maintains the peptide stability, sacrifices the regulatory control by the physiological system. (iii) The documented 24-aa MGF peptide is designed based on human MGF sequence. We used the full-length MGF of murine origin to avoid variation from species differences (Matheny et al., 2010; Rotwein, 2014; Vassilakos et al., 2014). For these reasons, we would prefer using the full-length MGF to MGF E-peptide analog to study the physiological effects of MGF.

Although MGF overexpression in skeletal muscle has been reported (Barton et al., 2010; Brisson et al., 2014), whether MGF regulates another IGF-1 splice form, IGF-1Ea, has not been examined. In this study, we found *Mgf* expression increased in muscle injury prior to *Igf-1Ea*, implying different regulatory mechanisms for the two IGF-1 isoforms. Furthermore, MGF overexpression did not alter *Igf-1Ea* expression in muscle and macrophages isolated from the injured muscle. Our study is the first to show that muscle IGF-1Ea expression is independent of MGF upregulation *in vivo*. The observed phenotypic changes resulted mainly from MGF upregulation.

The inflammatory response in muscle injury is comprised of several overlapping phases (Tidball and Rinaldi, 2012; Bentzinger et al., 2013), including initiation, perpetuation, and resolution. The initiation phase occurring immediately after injury involves the immediate recruitment of exudate myeloid cells into the injured muscle, mediated by resident macrophages (Brigitte et al., 2010). Given that *Mgf* was not significantly upregulated until 1 day post-injury (Lu et al., 2011b; Tonkin et al., 2015); it is unlikely that MGF contributes to the initiation phase.

During the perpetuation phase, myeloid cells, such as neutrophils and macrophages continue to infiltrate the injured muscle and enhance inflammatory signaling by secreting pro-inflammatory cytokines and chemokines (Stout et al., 2005; Tidball et al., 2014; Tidball, 2017). Our flow cytometric analysis revealed infiltration of macrophages following CTX-induced muscle injury beginning at 0 day and peaking at 3 days, coincided with previous reports (Radley and Grounds, 2006; Dumont et al., 2007; Brigitte et al., 2010; Lu et al., 2011a,b). *Tnf-α* and *Ccl2* expression were also elevated above baseline levels on 2 days post-injury. During the perpetuation phase of muscle inflammatory response, *Mgf* was significantly upregulated (Lu et al., 2011b; Tonkin et al., 2015) but MGF overexpression did not appear to affect infiltration of macrophages or the expression of inflammatory markers at this early inflammatory process.

One of the characteristics of the resolution phase is the clearance of pro-inflammatory myeloid cells, including neutrophils and pro-inflammatory macrophages (Gautier et al., 2013; Ortega-Gómez et al., 2013; Sciorati et al., 2016). As shown in our study, between 3 and 5 days post-injury, the number of pro-inflammatory macrophages decreased. At 4 days post-injury, we observed that MGF overexpression increased the number of pro-inflammatory macrophages, which coincided with increase of pro-inflammatory macrophage marker *Cd86*. The increase of macrophages at 4 days is in part caused by inhibiting macrophage apoptosis. Apoptosis is the major pathway for the clearance of pro-inflammatory macrophages during inflammatory resolution (Tidball and St Pierre, 1996; Horiguchi et al., 2002; Gautier et al., 2013; Sciorati et al., 2016). Previous studies have reported that MGF may protect against cell death in skeletal muscle (Barton et al., 2010). Also, MGF overexpression in skeletal muscle upregulates osteopontin, a potent mediator of macrophage activity and survival (Barton et al., 2010). This same study also showed that MGF overexpression upregulates expression of the main anti-apoptotic gene *Bcl-X*. Further work is needed to understand the mechanism by which MGF inhibits macrophage apoptosis.

Apart from clearance by apoptosis, the resolution of pro-inflammatory macrophages can occur by polarization into anti-inflammatory phenotypes (Tidball and St Pierre, 1996; Horiguchi et al., 2002; Gautier et al., 2013; Sciorati et al., 2016). In the MGF-overexpressing injured muscle, a transient increase in pro-inflammatory macrophage accumulation was observed at 4 days post-injury. Thereafter, the population returned to the level as the vector + CTX control group. During this time course, IGF-1 autocrine signaling typically polarizes macrophages into an anti-inflammatory phenotype. Reduced IGF-1 expression in macrophages has been shown to result in persistent infiltration of pro-inflammatory macrophages with a concurrent reduction in their anti-inflammatory counterparts (Tonkin et al., 2015). Furthermore, the upregulation of IL-10 by MGF overexpression implies a microenvironment in favor for anti-inflammatory polarization (Meador et al., 2008; Deng et al., 2012). Taken together, these two factors might explain why MGF overexpression only transiently increased the number of pro-inflammatory macrophages and did not appear to affect muscle regeneration outcomes. Clearly, interaction of macrophages with IGF-I and its isoforms are not the only regulatory factors for muscle regeneration. In our experiments, the peak of MGF expression did not coincide with the early infiltration of neutrophils in muscle injury suggesting that neutrophils may not be a target of MGF. Our *in vitro* data further shows that muscle cell is not directly involved in the induction of pro-inflammatory cytokine production in response to MGF overexpression. There are other cellular players (e.g., eosinophils, fibro-adipogenic progenitor cells, and regulatory T-cells) and derived molecules (e.g., IL-4 and IL-33) involved in the inflammatory resolution and the regeneration phase of muscle healing (Burzyn et al., 2013; Heredia et al., 2013; Sciorati et al., 2016; Schiaffino et al., 2017) but association of these cell types with MGF is not entirely clear. Of note, dynamics of inflammatory cells and the profiles of inflammatory cytokine vary between muscle fiber types upon muscle injury (Zimowska et al., 2017). It is possible that MGF differentially regulates inflammatory responses between muscles of unique fiber-type compositions.

We demonstrated the inhibitory effect of MGF overexpression on macrophage apoptosis in muscle injury. Macrophages seem to be the predominant source of MGF in muscle injury with the previous study (Tonkin et al., 2015). Our data suggests a role for autocrine/paracrine MGF signaling in modulating macrophage apoptosis in the resolution phase of inflammation in muscle injury. It would be interesting to further investigate the importance of MGF in macrophages by using a macrophage-specific MGF knockout model. However, data on the specificity and efficiency of macrophage-Cre lines are limited and questionable (McCubbrey et al., 2017). Alternatively, bone marrow (BM) transplantation experiments can be carried out using *Mgf^-^*^/^*^-^* mice and wild-type (WT) mice as BM donors and C–C motif chemokine receptor knockout (*Ccr2^-^*^/^*^-^*; with macrophages lacking the ability to infiltrate) mice as BM recipients (Saclier et al., 2013). The use of these models for transplantation experiment may shed light on the physiological roles of MGF in the macrophage-mediated inflammatory response of muscle injury.

One limitation of our overexpression model is that we were only able to identify the pre-pro-MGF peptide but not the putative secretory forms, i.e., pro-MGF or Eb-peptide. It has been shown that insertion of epitope tag at the C-terminal E-domain of MGF does not affect its release and take-up by cells *in vitro* (Pfeffer et al., 2009). This detection failure was probably due to MGF relatively short half-life (Brisson and Barton, 2012) and susceptibility to endopeptidase degradation (Pfeffer et al., 2009). Recently, an antibody against MGF identified pro-MGF and Eb-peptide *in vivo* using an optimized Western blot protocol (Vassilakos et al., 2017). Adaptation of this optimized protocol and acquisition of this antibody could confirm the presence of pro-MGF and Eb peptide in our MGF overexpression samples.

## Conclusion

In conclusion, our findings contribute to the understanding of the role of MGF in muscle injury. We identified macrophages as the major myeloid source of MGF in injured muscles. Our findings demonstrate (i) an increase in macrophage population in the MGF-overexpressing muscles compared to vector control after muscle injury; (ii) an upregulation of M1 macrophage markers as well as pro-inflammatory cytokines upon *Mgf* overexpression; and (iii) a reduction in the numbers of apoptotic macrophages in MGF-overexpressing muscles when compared to vector control. These together suggest that MGF overexpression may delay the resolution of pro-inflammatory macrophages that lead to the upregulation of inflammatory cytokines in muscle injury. Further studies on the mechanism of MGF apoptotic suppression in macrophages are needed. It would provide insights on the role of MGF signaling in pathological conditions in which macrophage is involved.

## Data Availability Statement

All datasets generated and analyzed for this study are included in the manuscript and the supplementary files.

## Author Contributions

K-TS performed the main experiments, data acquisition and analysis. All authors contributed to the conception and design of the work, interpretation of the results, writing, and editing of the manuscript.

## Conflict of Interest Statement

The authors declare that the research was conducted in the absence of any commercial or financial relationships that could be construed as a potential conflict of interest.
