# Supplementary material for: Overexpression of Mechano-Growth Factor Modulates Inflammatory Cytokine Expression and Macrophage Resolution in Skeletal Muscle Injury
Source: Front Physiol. 2018 Jul 26;9:999. doi: 10.3389/fphys.2018.00999 (PMC6094977; doi:10.3389/fphys.2018.00999)
Supplement: Supplementary file 1 [file Table_1.PDF]

Supplementary Table 1

	Gene of interest	*Assay ID*	*Gene ID*
**Housekeeping genes**	*Gapdh*	4352932E	NM_008084.2
	18S rRNA	4448484	X03205.1
	Rps20	Mm02342828_g1	NM_026147.5
**IGF-1 isoform**	*Igf-1Ea*	Mm00710307_m1	NM_001111275.1
	Mgf	Custom assay:<br>*Forward primer*<br>(5'-GACATGCCCAAGACTCAGAAGT-3')<br>*Reverse primer*<br>5" -CTTCTCCTTTGCAGCTTCGTTTT-3'<br>*Probe sequence*<br>MGB-FAM-5" -TCCCTATCGACAAACAAG-3"	
**Inflammatory cytokines**	*Tnf-α*	Mm00443258_m1	NM_013693.3
	Il-6	Mm00446190_m1	NM_031168.1
	Il-10	Mm01288386_m1	NM_010548.2
	Ccl2	Mm00441242_m1	NM_011333.3
**Macrophage surface antigen**	*Cd86*	Mm00444543_m1	NM_019388.3
	Cd206	Mm01329362_m1	NM_008625.2
**Satellite cells**	*Pax7*	Mm01354484_m1	NM_011039.2
**Myogenic regulatory factors**	*Myod*	Mm00440387_m1	NM_010866.2
	Myog	Mm00446194_m1	NM_031189.2
**Myosin heavy chain**	*Myh3*	Mm01332463_m1	NM_001099635.1
	Myh8	Mm01329494_m1	NM_177369.3

Supplementary Table 1: Taqman assays used in the present study. Manufacturer assay ID and Gene ID can be referred to Thermo Fisher Scientific, USA and GenBank, respectively. Taqman assay against MGF was designed via the custom Taqman assay design tool (Thermo Fisher Scientific, USA). The sequences of primer pairs and probe are indicated.
