# Supplementary figures and images for: Overexpression of Mechano-Growth Factor Modulates Inflammatory Cytokine Expression and Macrophage Resolution in Skeletal Muscle Injury
Source: Front Physiol. 2018 Jul 26;9:999. doi: 10.3389/fphys.2018.00999 (PMC6094977; doi:10.3389/fphys.2018.00999)

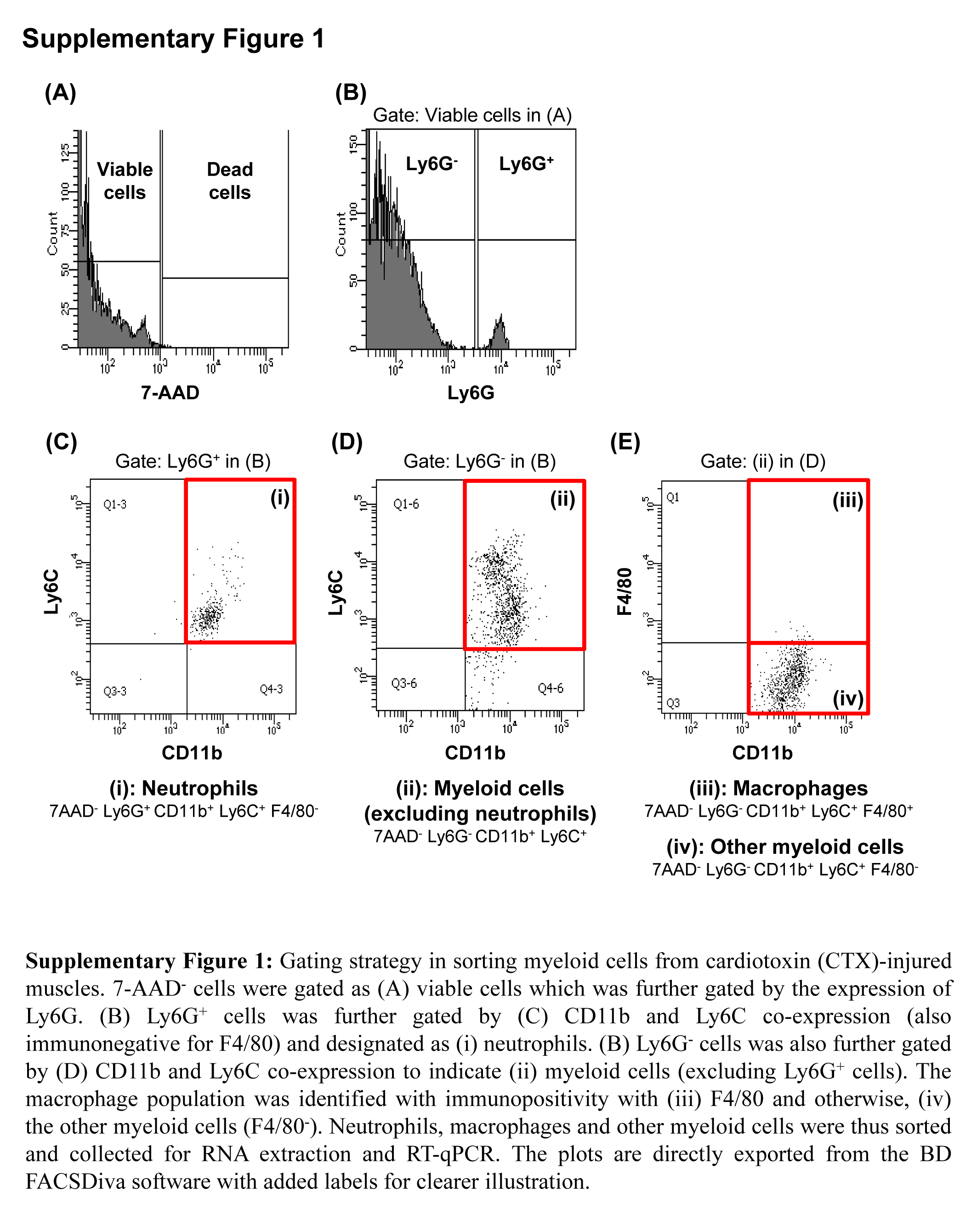

Supplement: Supplementary file 2 [file Image_1.TIF]

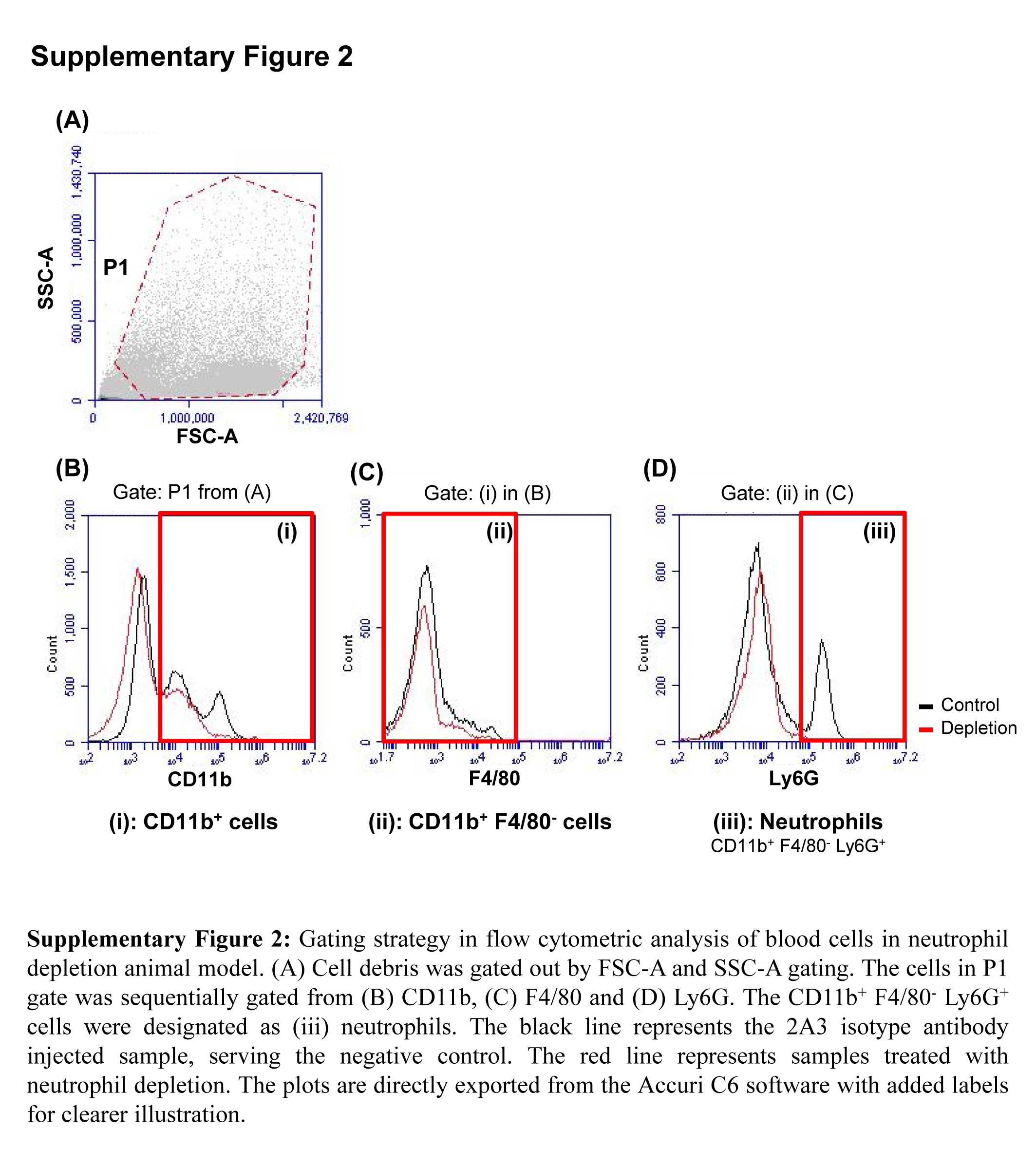

Supplement: Supplementary file 3 [file Image_2.TIF]

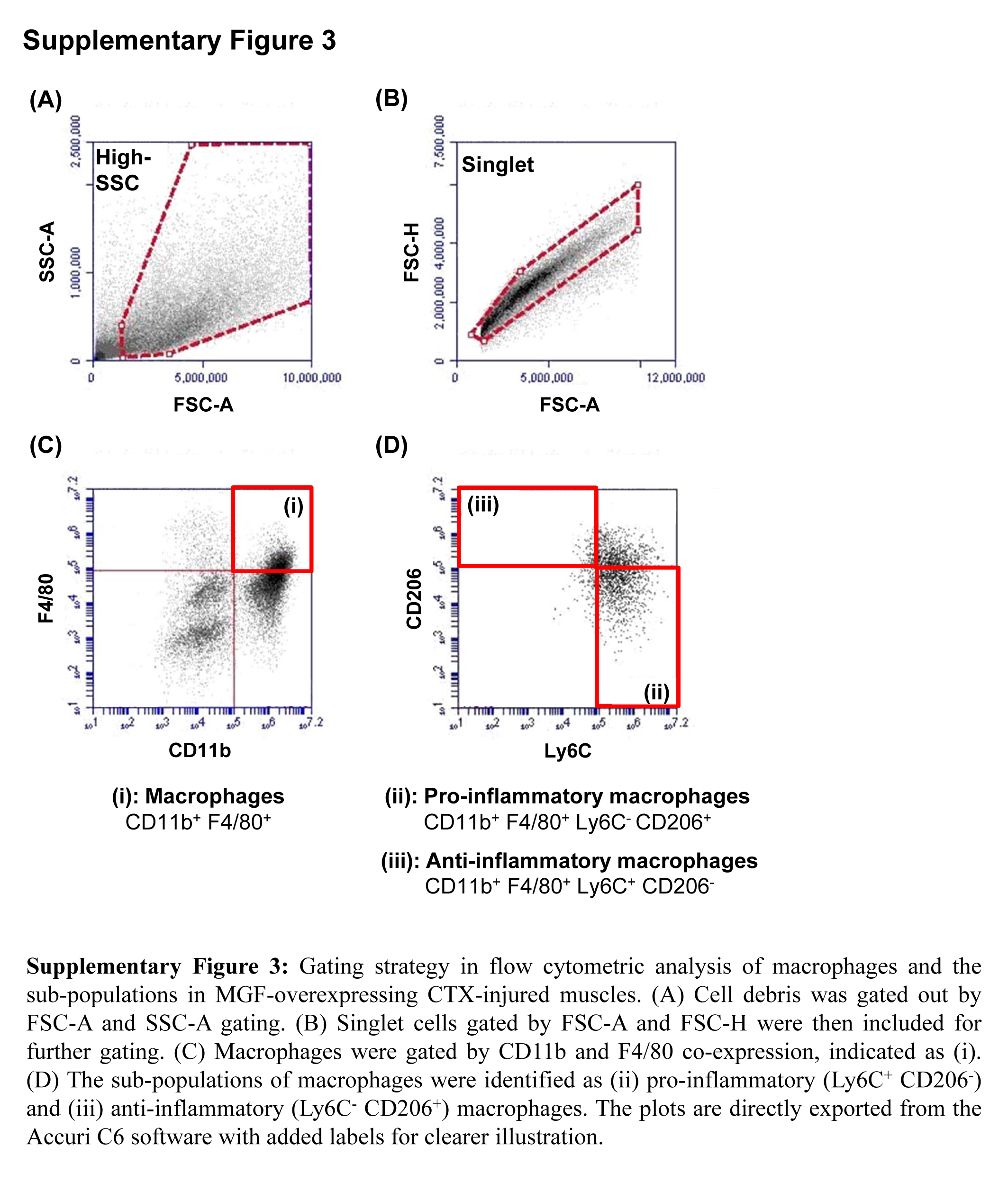

Supplement: Supplementary file 4 [file Image_3.TIF]

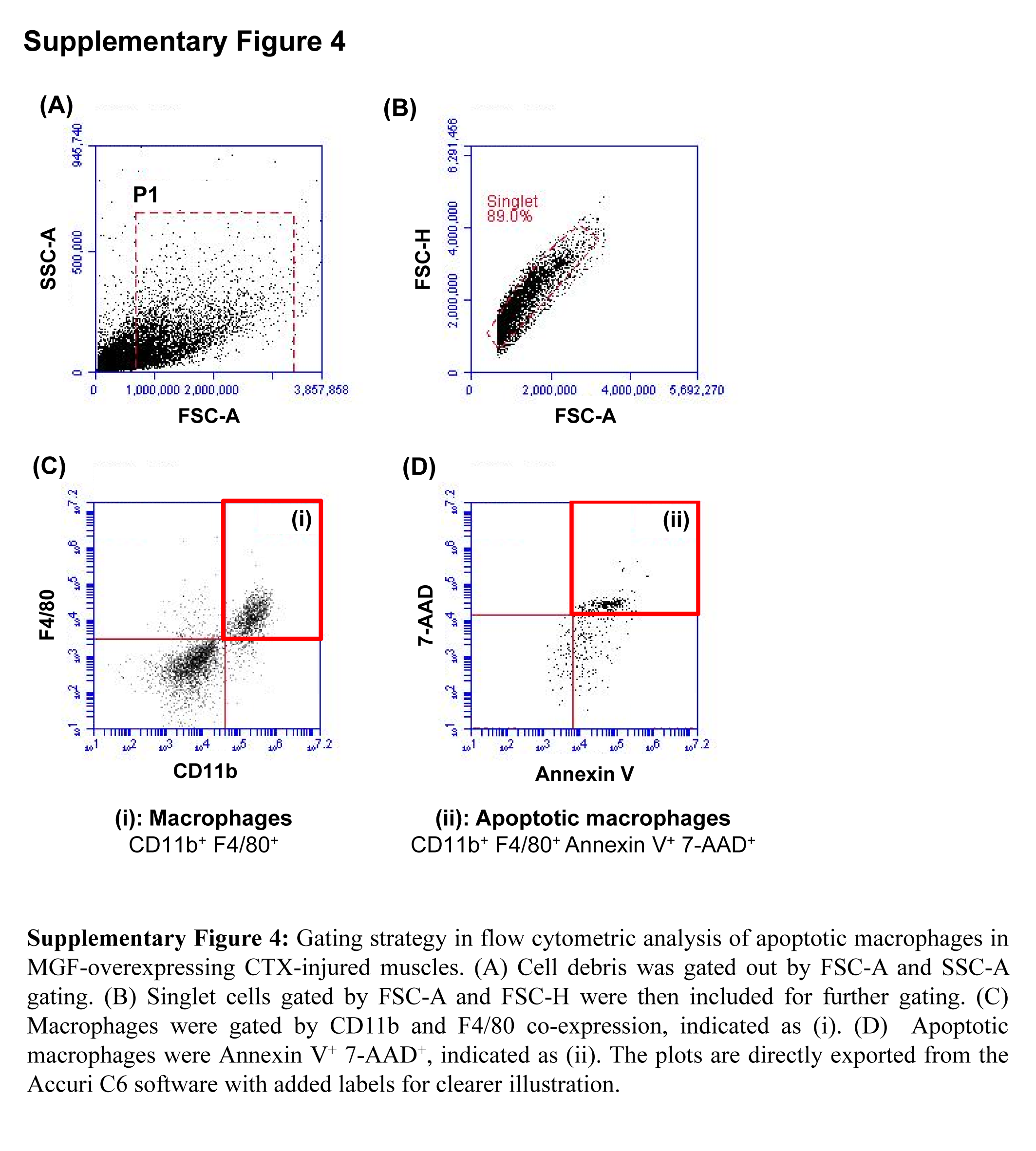

Supplement: Supplementary file 5 [file Image_4.TIF]
